# Identifying tests to evaluate in a diagnostic accuracy study for patients with vertigo in general practice: a Delphi study

**DOI:** 10.1186/s12875-025-02920-z

**Published:** 2025-08-02

**Authors:** Anna-Marie R. Leemeyer, Andrew K. Ross, Tjasse D. Bruintjes, Jochen W.L. Cals, Roeland B. van Leeuwen, Birgit I. Lissenberg-Witte, Vincent A. van Vugt, Otto R. Maarsingh

**Affiliations:** 1 Department of General Practice, Amsterdam UMC location Vrije Universiteit Amsterdam, de Boelelaan, Amsterdam, 1117 the Netherlands; 2 Amsterdam Public Health, Quality of Care, Amsterdam, the Netherlands; 3 Department of Otorhinolaryngology, Leiden University Medical Center, Leiden, the Netherlands; 4 Department of Otorhinolaryngology, Gelre Hospital Apeldoorn, Apeldoorn, the Netherlands; 5 Department of Family Medicine, Care and Public Health Institute (CAPHRI), Maastricht University, Maastricht, the Netherlands; 6 Department of Neurology, Gelre Hospital Apeldoorn, Apeldoorn, the Netherlands; 7 Department of Epidemiology and Data Science, Amsterdam UMC location Vrije Universiteit Amsterdam, de Boelelaan, Amsterdam, 1117 the Netherlands

**Keywords:** Dizziness, Vertigo, Delphi technique, Consensus, General practice, Primary Health Care

## Abstract

**Introduction:**

Vertigo is a common symptom that strongly impacts patients’ quality of life. More than 80% of patients experiencing vertigo are primarily treated by their general practitioner (GP). The GP’s'diagnostic toolkit' for vertigo has serious limitations, though, because diagnostic accuracy studies on conditions that may cause vertigo have never been performed in a general practice setting. Our aim was to determine which tests should be investigated in a diagnostic accuracy study for patients with vertigo in general practice.

**Method:**

We conducted an online Delphi procedure involving national and international experts. The experts were asked to judge a selection of 40 diagnostic tests based on the Dutch GP guideline on vestibular symptoms, supplemented by tests identified during a systematic review. Panellists were allowed to suggest additional tests after the first round. In case of consensus of at least 70%, a test was included or excluded. We also conducted a secondary sub-analysis of our Delphi procedure to demonstrate non-dominance of Dutch experts within our expert panel. Data were analysed using descriptive statistics and content analysis. Data were analysed using descriptive statistics and content analysis.

**Results:**

A panel of 20 experts from five countries, including 7 specialists in otolaryngology, 6 neurologists and 7 GPs, participated in the Delphi procedure. The panel judged 46 diagnostic tests in total, with 6 additional tests added to the original selection based on suggestions by experts. After the first two rounds (100% response rate), 16 tests were included, 22 tests were excluded and no consensus was reached on 8 tests. During the consensus round, one of the 8 tests was added to the included 16 tests. Of these 17 tests, 15 are recommended by the Dutch GP guideline, supplemented by the non-recommended Tandem walking test and the Romberg test.

**Conclusions:**

An international expert panel reached consensus on 17 tests for vertigo in general practice that should be investigated in a diagnostic accuracy study.

**Supplementary Information:**

The online version contains supplementary material available at 10.1186/s12875-025-02920-z.

## Background

The Bárány Society, the leading international organization for clinicians and researchers involved in vestibular medicine, developed a uniform way to describe vestibular symptoms. In the International classification of Vestibular Disorders (ICVD), vertigo is described as “the sensation of self-motion when no self-motion is occurring or the sensation of distorted self-motion during an otherwise normal head movement” [1, 2]. Vertigo is a common symptom that considerably affects patients’ quality of life [3–6]. Previous epidemiological studies report a prevalence of 5% in the adult population (18 or older), while another study reports a prevalence > 30% in the population aged 75 or older [3, 7]. The impact on patients is enormous: four out of five patients with vertigo report severely impairing symptoms, leading to medical consultation, interruption of daily activities, and/or avoidance of leaving the house which could lead to decreased productivity by work absenteeism [4, 8]. Since vestibular symptoms are strongly associated with age [3, 9], the personal and economic burden of vertigo will increase significantly in the future due to the ageing population [10].

Despite the fact that GPs treat the large majority of patients with vertigo, their ‘diagnostic toolkit’ to differentiate between vestibular diseases, such as vestibular neuritis, benign paroxysmal positional vertigo (BPPV), vestibular migraine and Meniere’s disease - as recommended by the Dutch GP guideline on vestibular symptoms [11], is seriously limited. There is no empirical evidence on the diagnostic value of history taking and physical examination for a patient with vertigo in general practice, because diagnostic accuracy studies on conditions that may cause vertigo have never been performed in a general practice setting [12]. Sufficient diagnostic test accuracy is crucial, because biased test results can lead to incorrect diagnosis and treatment [13]. Ultimately, we therefore aim to set up a large accuracy study for patients with vertigo in general practice [14]. To accomplish this, we need to start with a preselection in diagnostic tests. A Delphi procedure is a well-established approach to answer a research question by finding consensus when scientific evidence is absent or contradictory [15–18]. The aim of this Delphi procedure was to determine which tests should be investigated in a diagnostic accuracy study for patients with vertigo in general practice.

## Methods

### Sources of evidence

Prior to the Delphi procedure, we performed a comprehensive literature search in PubMed, Embase and Web of Science from January 2010 to March 2022. This time interval was chosen because Maarsingh et al. had already conducted an extensive search strategy prior to 2010 [19], in order to identify potentially relevant diagnostic tests for dizziness/vertigo in general practice (query search strategy: see Appendix I). We carried out the systematic screening of articles with the help of Covidence, an online platform for the management of systematic reviews [20]. Because of the number of studies identified, the title-abstract screening was conducted by nine screeners (six medical students and three researchers, i.e. AL, VV, OM). Title-abstract of each article was screened by two screeners. In case of disagreement, the article was discussed by AL, VV and OM to reach consensus. Full text screening was performed by two screeners (AL, VV or OM). The title-abstract screening of the original 13,737 articles resulted in the exclusion of 13,052 studies. We screened the remaining 671 for full text. Studies were excluded if the study did not concern a diagnostic accuracy study, the index test was not feasible in general practice, the index test was too expensive for general practice, the reference test was considered inappropriate (e.g. not relevant, not practical, inconsistency in the patient population), the study population did not include patients from a general practice or primary care setting, or the full text was not available in English, Dutch, German, or French. After full text screening, we included 119 relevant publications. Searching the reference lists of these publications did not yield additional relevant publication. The final 119 publications provided a total of 40 diagnostic tests of which 31 are also recommended by the Dutch GP guideline [11].

### Study participants

We predefined that the relevant specialisms of general practice, neurology and otorhinolaryngology each had to be represented by at least five panellists with demonstrable expertise on vertigo and/or dizziness, i.e. contribution to at least one vestibular guideline or at least 5 international peer reviewed publications related to vertigo and/or dizziness. We used a combination of expert judgment and snowball sampling to select the panellists. Through the research team’s professional network, we invited individuals based on their recognized expertise and involvement in the field, who could in turn nominate other experts. This led to a total number of invitations for 26 national and international experts on vestibular symptoms such as vertigo and dizziness, including seven GPs, nine neurologists and ten otorhinolaryngologists. Out of these 26 experts, 20 decided to take part in our Delphi procedure. Reasons for non-participation were lack of time (*N* = 2) or no response (*N* = 4). Informed consent was obtained from all participants by e-mail.

### Design

#### The Delphi method

A Delphi procedure concerns a series of sequential questionnaires presented in two or more rounds, interspersed with controlled feedback, in order to obtain the most reliable consensus from an expert panel [21, 22]. An important characteristic of the procedure is to resolve disagreement in a structured, predefined way by giving repeated and controlled feedback. A Delphi procedure is performed anonymously (to avoid dominance of members of the expert panel) and consists of minimally two rounds. After the first round the results are summarized and presented as feedback in the second round.

The main goal of a Delphi procedure is to establish a level of consensus; however, there is not a universally agreed-upon consensus threshold [21]. In Delphi studies, consensus is typically considered to be achieved when there is a substantial level of agreement, such as 70% or higher [22].

#### Delphi survey rounds

In the first round, each panellist received a score form with 40 potential diagnostic tests that should be assessed. The score form was accompanied by two documents, one document providing background information on the Delphi procedure (Appendix II), and another document with empirical evidence and background information– including links to instruction videos, if applicable– of each test (Appendix III). This list of tests was based on a systematic review, see paragraph “source of evidence” and the Dutch DP guideline [11]. The panellists received a score form by email, on which they could indicate if a particular test should be evaluated in a diagnostic accuracy study in general practice (yes/no). Panellists had to motivate why a test should be excluded from the diagnostic accuracy study. The score form contained six predefined categories for motivation of exclusion. These categories were derived from a framework often used to evaluate diagnostic technologies by categorizing studies into six hierarchical level: (1) technical feasibility, (2) diagnostic accuracy, (3) diagnostic thinking impact, (4) therapeutic choice impact, (5) patient outcome impact, and (6) societal impact [23].

If a panellist deemed that an important diagnostic test was missing, he or she could name this test on the score form with a brief description and motivation why it should be added. The researchers assessed each new test suggested by a panellist, examining whether the test was applicable in general practice, including considerations such as financial feasibility.

Each participant completed and returned the score form within six weeks by email. The researchers summarized the results. Tests on which at least 70% of the panellists agreed were either directly included in the diagnostic accuracy protocol or deleted from the list. The frequency of the responses per item was calculated and motivations were summarized per category per test. Tests on which no agreement had been reached (agreement level below 70%) were put on a new list, with a summary of motivations per test. A new score form for the second round was made for the panellists.

Panellists received information on [a] the percentage agreement per item in the first round, [b] the frequency of motivation categories, [c] a brief summary of comments and [d] an overview of their own scores compared to the group scores. In the second round each participant had to indicate for each remaining test if and why the test should be incorporated in the diagnostic accuracy protocol. This round offered the opportunity for panellists to change their score in view of the group’s response. The panel received a summary of the results. Again, tests on which at least 70% of the panellists agreed were either included in the protocol or deleted from the list. If there were tests on which no agreement had been reached (threshold 70%), additional rounds could be held if necessary.

#### Sensitivity analysis

In order to assess the potential consequences of the Dutch majority in our Delphi study (see Table 1), we performed a secondary sensitivity analysis of the first Delphi round using an expert subpanel consisting of non-Dutch international experts. Herein we compared the included and excluded tests after the first round between the expert subpanel and the full expert panel.

## Results

### Expert panel

We included 20 experts from five different countries with an average of 22 years of working experience (see Table 1). The response rate of participating panellists throughout the subsequent Delphi rounds was 100%.

**Table 1:** Characteristics of the Delphi panellists (*n* = 20)

*Characteristic*	*N*
*Sex*	
Female	7
Male	13
Age in years	Mean = 50 [range: 34–72]
*Occupation*	
General practitioner	7
ENT specialist	7
Neurologist	6
*Location of panellists*	
The Netherlands	11
United Kingdom	4
United States of America	3
Belgium	1
Australia	1
*Years of working experience*	Mean = 22.4 [range: 5–45]
*Numbers of international publications on vertigo*	Mean = 19 [range: 0–70]

### Delphi rounds

The final results of the Delphi procedure are shown in Tables 2 and 3. After the first round, 15 out of 40 tests were included, 11 tests were excluded and 14 tests did not reach agreement. The most mentioned reasons for exclusion were technical feasibility, (lack of) diagnostic accuracy and diagnostic thinking impact. During this round the panel suggested 10 additional tests to assess during the next round. Four of these tests were not included in the second round, i.e. portable video-oculography (VOG), was deemed too expensive for use in general practice, while the other three tests were already on the list.

**Table 2:** Results Delphi procedure: included tests

Diagnostic Test *N* = 40 + 6	Round one					Round two				Consensus round
	Inclusion	Exclusion	No Expertise	Most important exclusion motivation,	Result^#^	Inclusion	Exclusion	No Expertise	Result, Ω	Result, Ω
**I. Patient history** [11]										
1. Present Vertigo Symptoms	19	1	0	b, c, d, e, f	I					
2. Accompanying symptoms	19	1	0	-	I					
3. Timing of symptoms	20	0	0	-	I					
4. Triggers of symptoms	19	1	0	-	I					
5. Medication	18	2	0	b, c	I					
6. Past medical history	20	0	0	-	I					
7. Cardiovascular risk factors	17	2	0	b, c, d, e	I					
**II. Physical examination**										
8. Pulse and blood pressure [24–26]	18	2	0	b, c	I					
9. Otoscopy [11, 27, 28]	18	2	0	a, b	I					
10. Audiometry [29–31]	10	14	0	a, c, d, e, f	Round two	5	15	0	E	
11. Motor and Sensory function (Global neurological exam) [13, 32, 33]	19	1	0	a, b	I					
12. Nystagmus, general observed [11, 34–37]	18	2	0	a, c	I					
13. Head-shaking nystagmus [31, 36, 38–40]	4	16	0	a, b, c, d, e	E					
14. Head Impulse-Nystagmus-Test of Skew (HINTS) [41–52]	10	10	0	a, b, c, d, e	Round two	7	12	1	Consensus round	I
Alternative HINTS Plus						7	12	1	Consensus round	I
15. Sideways stepping/Fukuda Stepping/Unterberger test [40, 53–59]	6	14	0	a, b, c, d, e, f	Round two	2	17	1	E	
16. Dix-Hallpike test [40, 60–67]	20	0	0	-	I					
17.Head-roll/Supine-roll test [11, 68–70]	14	6	0	a, b, c, d, e	I					
18. STANDING algorithm (SponTAneous Nystagmus, Direction, head Impulse test, standing) [48, 71–73]	9	10	1	a, b, c, d, e, f	Round two	5	15	0	E	
19. Gait disorientation test [74–76]	1	19	0	a, b, c, d, e, f	E					
20. Tandem walking test [53, 77–81]	16	4	0	a, b, c, d, f	I					
21. Bucket test [37, 82–86]	2	17	0	a, b, c, d, e, f	E					
22. Romberg test[39, 77, 78, 81, 87, 88]	15	5	0	c, f	I					
23. Single leg stance test [77]	4	16	0	a, b, c, d, f	E					
24. Timed up & Go test [59, 77]	5	15	0	a, b, c, d, f	E					
25. Past pointing test [59]	11	8	1	b, c, d, e, f	Round two	7	13	0	Consensus round	E
26. Five Times Sit to Stand test [59, 89]	2	18	0	a, b, c, d, e, f	E					
27. Pull test [59]	2	18	0	a, b, c, d, e, f	E					
28. Frenzel glasses [90]	9	11	0	a, b, c, d, e	Round two	9	11	0	Consensus round	E
**III. Additional tests**										
29. Laboratory testing [91–104]	5	14	0	a, b, c, d, e, f	E					
**IV. Questionnaires**										
30. PCI Score System [105]	6	12	2	a, b, c, d, e	Round two	5	13	1	E	
31. Essen Score System [105, 106]	2	14	0	a, b, c, d, e	E					
32. ABCD^2^ Score System [37, 43, 44, 48, 52, 105, 107, 108]	3	14	0	a, b, c, d, e	E					
33. TriAGe + Score System [107]	4	12	0	a, b, c, d, e	E					
34. Questionnaires Benign Paroxysmal Positional Vertigo	10	9	1	a, b, c, d,e	Round two					
Chen et al. [109]						5	15	0	E	
Kim et al. [110]						5	15	0	E	
Lapena et al. [111]						2	18	0	E	
35. Questionnaire Vestibular Migraine [112]	9	10	1	a, b, c, d, e	Round two	8	12	0	Consensus round	E
36. Questionnaires Vestibular Disorders	6	13	1	a, b, c, d, e	Round two					
Steward et al. [113]						3	17	0	E	
Bayer et al. [114]						2	18	0	E	
**V. Additional tests suggested by Delphi Panel**										
I. Romberg with Jendrasik [77]						5	18	2	E	
II. Orthostatic blood pressure [115]						17	2	1	I	
III. Dynamic Visual Acuity [116, 117]						4	14	2	E	
IV. Carmona Gait Ataxia Severity Score [45]						6	12	2	Consensus round	E
V. Tuning Forks [118]						11	8	1	Consensus round	E
VI. Electrocardiogram (ECG) [119]						9	8	2	Consensus round	E

a: Technical feasibility; b: Diagnostic accuracy; c: Diagnostic thinking impact; d: Therapeutic choice impact; e: Patient outcome impact; f: Societal impact

# = I: Inclusion, E: Exclusion

**Table 3:** Final selection of diagnostic tests which should be investigated in a diagnostic accuracy study^a^

**I. Patient history**	**Description**
1. Present vertigo symptoms	What kind of vertigo symptoms does the patient experience?
2. Accompanying symptoms	Does the patient experience other symptoms associated with vertigo symptoms (e.g. nausea, vomiting, tinnitus)?
3. Timing of symptoms	When does the patient experience his/her complaints of vertigo? Is it a constant feeling or are there attacks of vertigo? What is the duration of attacks (e.g. seconds, minutes, hours)? How often does it occur (daily, weekly, monthly)?
4. Triggers of Symptoms	Does the patient describe activities/factors that provoke the vertigo (e.g. bending over, looking up, loud noises)?
5. Medication	Does the patient use medication? Does he/she smoke or use alcohol or drugs?
6. Past medical history	Does the patient have a medical history of cardiovascular diseases, internal diseases, ENT diseases, neurological diseases, musculoskeletal diseases, and/or visual diseases?
7. Cardiovascular risk	Does the patient have known cardiovascular risk factors (e.g. smoking, obesity, high blood pressure)?
**II. Physical examination**	
8. Pulse and blood pressure	Pulse measurement and blood pressure measurements can be performed to assess the hemodynamic condition of a patient. Hemodynamic abnormalities can be associated with vestibular symptoms.
9. Otoscopy	Otoscopy can be used to diagnose inflammation/infection of the inner ear, perforation of the tympanic membrane, perilymphatic fistula or cholesteatoma. These diagnoses can be associated with vestibular symptoms.
10. Global neurological exam	The standard neurological clinical assessment is used to evaluate the nervous system. This exam is used to diagnose disorders affecting the brain, nerves and spinal cord that may cause vestibular symptoms.
11. Nystagmus, general observed	A nystagmus is an involuntary eye movement, that is directly followed by a compensatory rapid eye movement in the contradictory direction. The occurrence of nystagmus can be associated with vestibular symptoms and problems within the vestibular system.
12. HINTS (Head Impulse-Nystagmus-Test of Skew)	The HINTS exam consists of the Head Impulse Test (HIT), Nystagmus examination and Test of Skew. The HIT is also known as the Head Thrust Test (HTT). The HINTS is a clinical bedside test that is used to differentiate peripheral vestibular disease from stroke in patients with an acute vestibular syndrome (AVS).
13. Dix-Hallpike	The Dix-Hallpike manoeuvre is a clinical test for benign paroxysmal positional vertigo (BPPV) of the posterior semicircular canal.
14. Head-roll/Supine-roll test	The Head-roll test is a clinical test for benign paroxysmal positional vertigo (BPPV) of the lateral (horizontal) semicircular canal.
15. Tandem walking test	The Tandem walking Test is a clinical test used to screen patients for neurologic and vestibular disorders.
16. Romberg test	The Romberg test is a clinical test used to assess the dorsal columns of the spinal cord and the general balance of the patient. Vestibular dysfunction could be accompanied by balance problems.
17. Orthostatic blood pressure test	An abnormal fall in blood pressure on standing from a supine or sitting position has been described as orthostatic hypotension. Orthostatic blood pressure measurement could be performed for patients who complains of dizziness or syncope.

^a^For background information see Appendix III

Round two started with 20 tests to be assessed, i.e. 14 tests remaining from the first round and six tests added by the panel. After round two, one test was included and 11 were excluded. The panel could not reach consensus on eight tests, namely Head Impulse-Nystagmus-Test of Skew (HINTS), HINTS Plus, Past Pointing Test, Frenzel glasses, Questionnaire Vestibular Migraine, Dynamic Visual Acuity, Tuning forks and ECG.

In total 16 test were included after two rounds.

Given the test scores of round two (33-58%) and the respective comments, it was unlikely that one of the remaining eight tests would reach the threshold of ≥ 70% during another Delphi round. Despite not reaching the threshold, the research group decided to add the HINTS Plus (not structural, but on indication) to the set of 16 index tests, because: (1) the high level of empirical evidence for the application of the HINTS [120] is incomparable to the evidence for other tests that did not reach the inclusion threshold; (2) the HIT (not structural, but on indication) is already recommended in the Dutch GP guideline, which warrants verification of its accuracy in a general practice setting; (3) some panellists disputed the ability of non-experts or GPs to perform and interpret the HINTS, but this was never verified.

During a 7-day timeframe, panellists were invited to raise objections to the draft protocol, including the inclusion of the HINTS test. No objections were raised during this period.

Table 3 describes the final list of 17 diagnostic tests to be investigated in a diagnostic accuracy study for patients with vertigo in general practice.

At the end of this Delphi procedure, the expert panel selected 17 tests to be investigated in a diagnostic accuracy study. The panel endorsed 15 tests as recommended by the Dutch GP guideline on vestibular symptoms [11], supplemented by the Tandem walking test [26, 53, 77, 78] and the Romberg test [77, 78, 87, 88].

### Sensitivity analysis

Using the subpanel consisting of our 9 international experts, we found quite similar results to those of the full expert panel after the first round (see Table 4). More specifically, within the subpanel 14 tests were included after the first round, all of which were also in the 15 tests included after the first round within the full panel (93%). In particular, only one test, the Head-roll/Supine Roll test, scored insufficiently (56%) within the subpanel and so would go on to the second round instead of directly being included as it did after the first round in the full panel.

**Table 4:** Sensitivity analysis: comparison of the full expert panel results to the expert subpanel results after the first round

	Full panel (*n* = 20)			Subpanel (*n* = 9)		
Tests	Inclusion: yes > 70%	Exclusion: yes < 30%	Round 2: yes = 30 to 70%	Inclusion: yes > 70%	Exclusion: yes < 30%	Round 2: yes = 30 to 70%
**I. Patient history**						
1. Present vertigo symptoms	95%			89%		
2. Accompanying symptoms	95%			89%		
3. Timing of symptoms	100%			100%		
4. Triggers of symptoms	95%			89%		
5. Medication	90%			78%		
6. Past medical history	100%			100%		
7. Cardiovascular risk	85%			89%		
***II. Physical examamination***						
8. Pulse and blood pressure	90%			89%		
9. Otoscopy	90%			89%		
10. Audiometry			30%		22%	
11. Motor and sensory function (global neurological exam)	95%			89%		
12. Nystagmus, general observed	90%			100%		
13. Head-shaking nystagmys		20%			22%	
14. HINTS (Head Impuls, Nystagmus, Test of Skew)			50%			44%
15. Sideways Stepping/Fukuda Stepping/Unterberger Test			30%		22%	
16. Dix-Hallpike	100%			100%		
17. Head-roll/Supine-roll Test	70%					56%
18. STANDING algorithm (SponTAneous Nystagmus, Direction, head Impuls test, standiNG)			45%			44%
19. Gait Disorientation Test		5%			0%	
20. Tandem Walking Test	80%			100%		
21. Bucket Test		10%			0%	
22. Romberg Test	75%			100%		
23. Single Leg Stance Test		20%			22%	
24. Timed up & Go Test		25%				33%
25. Past Pointing Test			55%			56%
26. Five Times Sit to Stand Test		10%			0%	
27. Pull Test		10%			0%	
28. Frenzel glasses			45%			44%
***III. Additional Tests***						
29. Laboratory testing		25%				56%
***IV. Questionnaires***						
30. PCI Score System			30%		22%	
31. Essen Score System		10%			11%	
32. ABCD^2^ Score System		15%			22%	
33. TriAGe + Score System		20%			11%	
34. Questionnaires Benign Proxysmal Positional Vertigo			50%			44%
35. Questionnaire Vestibular Migraine			45%			56%
36. Questionnaire Vesstibular disorders			30%			33%
TOTALS:	Yes: *N* = 15	No: *N* = 11	R2:*N* = 10	Yes: *N* = 14	No: *N* = 12	R2:*N* = 10

For the tests excluded in the first round of the subpanel we also find a substantial degree of agreement with the tests excluded after the first round of the full analysis. In particular, we find that of the 12 tests excluded after the first round in the subpanel, 9 of these were also excluded in the 11 tests excluded after the first round of the full panel (81%). In particular, within the subpanel, the Sideways Stepping test and PCI scoring were now outright excluded, while laboratory testing went onto the second round. There were no other discrepancies.

## Discussion

### Summary

We conducted a Delphi procedure to determine which tests should be investigated in a diagnostic accuracy study for patients with vertigo in general practice. Through the Delphi method, a structured and iterative approach for building consensus among experts from different specializations, we combined empirical evidence with expert opinion to formulate a definitive protocol for a diagnostic accuracy study. After the completion of the Delphi procedure, the international experts reached consensus on 17 tests for vertigo in general practice that should be further investigated in a diagnostic accuracy study [14]. The final test battery agreed upon contains 15 tests that were already recommended by the Dutch GP Guideline, supplemented by the non-recommended Tandem walking Test and the Romberg Test.

Our sensitivity analysis demonstrated substantial agreement between the subpanel and the full panel on both included (14 out of 15, i.e. 93%) and excluded tests (9 out of 11, i.e. 81%), suggesting that the majority of Dutch experts did not influence the study results in an important manner. A more likely explanation for the significant overlap between the included test battery by our panel and the Dutch guideline may therefore be that the guideline adequately reflects the international consensus on diagnosing vertigo in general practice.

Moreover, while checking the agreement between this guideline and the general international expert consensus was not a primary aim of this study, it is nonetheless an interesting finding and raises two natural questions. First, it might be interesting to see whether the large degree of consensus between the subpanel and the full panel holds with the addition of more international experts. If it does, then the applied Delphi method could in general be utilized as a novel kind of accuracy study methodology in order to gauge agreement between a country’s national guideline(s) and the international expert consensus on particular topics within primary care and secondary/tertiary care, or agreement between different specialties within secondary/tertiary care.

### Strengths and limitations

A strength of this study is the use of an extensive systematic literature search to identify potentially relevant diagnostic tests for vertigo in general practice. These tests were supplemented by a comprehensive appendix (see Appendix III), that explained each test, provided links to instructional videos, and references to supporting literature. Several experts commented that they found this appendix to be very valuable, as it facilitated completing of the scoring form during the Delphi procedure. Another strength of this study is the 100% response rate among the expert panel during the Delphi rounds [116]. A final strength was that our panel demonstrated substantial confidence in their own knowledge and expertise regarding tests for vertigo in general practice. They only stated ‘insufficient expertise’ in 2.6% of all judgments were stated as ‘insufficient expertise’, whereas this percentage was much higher during a previous, similar Delphi procedure (14.4%) [19]. One panellist suggested to add portable video-oculography (VOG) to the test selection [121, 122]. Unfortunately, this was not possible, because VOG is currently still too expensive and therefore not yet feasible for use in general practice.

A possible limitation of our study is that our strategy for the purposive sampling of experts based on expert judgement and snowball sampling might be too stringent. A less stringent threshold for judging expertise, i.e. less heavily relying on relevant academic or guideline publications, might result in a larger pool of experts to choose from.

Another possible limitation is that we have prematurely excluded non-academic clinical experts, e.g. experienced GPs and/or neurologists/ENT-specialists who in practice may have a generally recognized degree of clinical expertise on these topics, while not necessarily having any documented publications on these topics [123]. Trying to incorporate such non-academic clinical experts with undocumented specialized knowledge into a Delphi study poses significant challenges. Exactly due to lack of proper documentation, searching by random or using registry-based sampling would be problematic, where one is forced to rely on word-of-mouth and folk wisdom. Choosing to include such non-academic clinical experts would likely result in a larger pool of panellists, but with the trade-off of an unintended probable increase in the frequency of ‘insufficient expertise’ getting reported (or not) by the panellists with respect to the tests.

### Comparison with existing literature

We conducted a PubMed search to identify comparable studies. This search yielded a total of 13 articles, eight of which were identified as Delphi studies; one specifically addressed diagnostic tests for patients with dizziness. Maarsingh et al. [19] focused on tests for evaluating dizziness in older patients, while we focused on adults with vertigo. Despite similarities in history-taking importance and physical examination components, their study had a higher uncertainty rate (14.4%) compared to ours (2.6%), likely due to differing expert compositions. Maarsingh included additional tests like ECGs and questionnaires, not considered in our study, possibly due to differences in target populations and intended scope.

### Implications for research and practice

The 17 tests that were recommended in this Delphi procedure are currently being tested in a large diagnostic accuracy study called the Vertigo Diagnosis (VERDI) study (start recruitment March 2023; Trial registration number: ISRCTN97250704; Registration date: March 16th 2023; 10.1186/ISRCTN97250704) [14]. Currently, GPs have to diagnose patients with vertigo using a diagnostic toolkit that lacks scientific justification. By conducting this diagnostic accuracy study, a diagnostic algorithm can be developed to help GPs more accurately and efficiently identify an underlying cause in patients with vertigo, which may lead to faster efficient treatment, and an improvement of the overall outcome for patients with vertigo in general practice.

## Conclusion

Based on an international Delphi procedure, experts recommended 17 diagnostic tests for patients with vertigo in general practice to be evaluated in a diagnostic accuracy study, of which 15 tests were already recommended by the Dutch GP guideline, supplemented by the non-recommended Tandem Walking Tests and the Romberg Test.

## Supplementary Information

Supplementary Material 1.

Supplementary Material 2.

Supplementary Material 3.

## Data Availability

The dataset(s) supporting the conclusions of this article, including the individually completed score forms, are not publicly available due to confidentiality agreements with the participating experts. However, they are available from the corresponding author upon reasonable request, subject to maintaining the anonymity and confidentiality of the experts.

## Abbreviations

ECG: Electrocardiogram
GP: General Practitioner
HIT/HTT: Head Impulse Test / Head Thrust Test
HINTS: Head Impulse-Nystagmus-Test of Skew
ICVD: International Classification of Vestibular Disorders
BPPV: Benign Paroxysmal Positional Vertigo
VERDI study: Vertigo Diagnosis Study
VOG: Vestibular Oculography
