# Supplementary material for: Identifying tests to evaluate in a diagnostic accuracy study for patients with vertigo in general practice: a Delphi study
Source: BMC Prim Care. 2025 Aug 2;26:238. doi: 10.1186/s12875-025-02920-z (PMC12318412; doi:10.1186/s12875-025-02920-z)
Supplement: Supplementary file 1 — Supplementary Material 1. [file 12875_2025_2920_MOESM1_ESM.pdf]

Delphi procedure: an introduction

The VERtigo Diagnosis (VERDI) study

DEPARTMENT OF GENERAL PRACTICE, AMSTERDAM UMC, THE NETHERLANDS

Drs. A.R. Leemeyer, MD, PhD student
Dr. VA van Vugt, GP, Postdoctoral Researcher and Epidemiologist
Dr. O.R. Maarsingh, GP, Associate Professor and Epidemiologist
Contact: a.leemeyer@amsterdamumc.nl

A Delphi procedure: which tests should be investigated in a diagnostic accuracy study for patients with vertigo in general practice?

INTRODUCTION

Vertigo is a common symptom. The impact on patients is enormous: four out of five patients with vertigo report severely impairing symptoms, leading to sick leave, medical consultation, interruption of daily activities, and/or avoidance of leaving the house. The economic burden of vertigo is substantial, due to repeated and ill-targeted consultations, excessive use of diagnostic imaging, emergency care, and decreased productivity caused by work absenteeism [1]. It is expected that the population aged 65 years and over will increase from 9.3% to 16% in 2050 [2]. Since the prevalence of vestibular symptoms is strongly associated with age, the personal and economic burden of vertigo may increase significantly in the future.

More than 80% of patients experiencing vertigo in the Netherlands, United Kingdom, and United States are primarily treated by their general practitioner/primary care physician (GP/PCP) and are never referred to a medical specialist. The symptoms of these patients are predominantly caused by peripheral vestibular disease, like vestibular neuritis, benign paroxysmal positional vertigo (BPPV), Meniere's disease or vestibular migraine. Incidentally, vertigo in general practice has a central cause such as a cerebrovascular event. Despite the fact that GPs treat the large majority of patients with vertigo, their 'diagnostic toolkit' - as recommended by the revised Dutch Guideline on Dizziness [3] - is seriously limited. There is no empirical evidence on the diagnostic value of history taking and physical examination for patients with vertigo in general practice, because diagnostic accuracy studies on conditions that may cause vertigo have never been performed in a primary care setting. Sufficient diagnostic test accuracy is crucial, because biased test results can lead to incorrect diagnosis and treatment.

Recently, our research group received funding to perform an extensive diagnostic accuracy study on vertigo in general practice, the VERtigo Diagnosis (VERDI) study. The main objective of VERDI is to investigate the diagnostic accuracy of history taking and physical examination for patients with vertigo in general practice, in order to construct an easy-to-use diagnostic algorithm for daily clinical practice. Preliminary to the actual accuracy study we want to conduct a Delphi procedure to determine which tests should be investigated.

A Delphi procedure is a consensus procedure often used in the development of guidelines. The procedure can be used if scientific evidence is absent or contradictory [4, 5]. The Delphi procedure is a series of sequential questionnaires presented in two or more rounds, interspersed with controlled feedback, in order to obtain the most reliable consensus from an expert panel [6]. An important characteristic of the procedure is to resolve disagreement in a structured, predefined way by giving repeated and controlled feedback. A Delphi procedure consists of minimally two rounds. After the first round the results are summarized and presented as feedback in the second round.

AIM (of the present study)

To determine which diagnostic tests should be investigated in a diagnostic accuracy study for patients with vertigo in general practice.

METHOD

Design

We will conduct a Delphi procedure by e-mail.

Participants
We have invited national and international experts in the fields of general practice (n=7), neurology (n=7) and otorhinolaryngology (n=7). All participants have specific expertise on vertigo/dizziness, based on international peer reviewed publications and/or contribution to respective guidelines.

First Delphi round
In the first round, each participant will receive a score form with a diagnostic tests that should be assessed. This list of tests is based on an extensive literature search (see Appendix I) and the Dutch Guideline on Dizziness [6]. The tests are described in Appendix II. The participants receive a score form by e-mail, on which they can indicate if a particular test should be incorporated in the diagnostic protocol (yes/no). Participants should motivate why a test should be excluded from the diagnostic protocol. On the score form we will supply six predefined categories for motivation of exclusion. These categories were derived from a framework often used to evaluate diagnostic technologies by categorizing studies into six hierarchical levels, namely technical feasibility, diagnostic accuracy, diagnostic thinking impact, therapeutic choice impact, patient outcome impact, and societal impact [7] – see also the explanation at page 4.
If a participant thinks that a relevant test has been omitted, this can be registered on the score form with a brief description and motivation in the row “Missing tests”.
Each participant completes and returns the score form within 6 weeks by e-mail.

Assessments of the results
In the next two weeks the researchers summarize the results. Tests on which at least 70% of the participants agree will be either included in the protocol or deleted from the list. The frequency of the responses per item will be calculated and motivations will be summarized per category for each test. Tests on which no agreement has been reached (threshold 70%) will be put on a new list, with a summary of motivations per test.

Second Delphi round
Items not reaching the threshold of 70% agreement are presented in the second round to the participants. Participants receive information on [a] the percentage agreement per item in the first round, [b] the frequency of motivation categories, [c] a brief summary of comments, and [d] an overview of the own scores compared to the group scores. Participants also receive a list of tests on which at least 70% agreement has been reached. In the second round each participant has to indicate for each remaining test if and why the test should be incorporated in the diagnostic protocol. This round offers the opportunity for participants to change their score in view of the group’s response. Each participant completes and returns the score form within 2 weeks by e-mail.

Assessments of the results
In the next two weeks the researchers summarize the results. Again, tests on which at least 70% of the participants agree will be either included in the protocol or deleted from the list. Tests on which no agreement has been reached (threshold 70%) will be put on a new list, with a summary of motivations per test. If necessary a third round will follow in which participants only have to accept or reject the remaining tests on the list, without giving any motivation.

Next step
The selected diagnostic tests will be investigated in our diagnostic accuracy study.

EXPLANATION OF MOTIVATIONAL CATEGORIES ON THE SCORE FORM

1. Technical feasibility
Technical feasibility refers to the feasibility of a diagnostic test in daily clinical practice. Tests requiring a lot of time, knowledge, abilities or devices are often not feasible in general practice. In most practices, a consultation of maximally 10-20 minutes is booked per symptom/complaint. Also, the practice space is often limited. If there is a test of which you are not sure whether it is feasible in general practice, you can put an X in the respective column. Please elaborate on your doubts in the column for comments.

2. Diagnostic accuracy
When diagnostic accuracy measures are calculated/estimated incorrectly, this can lead to wrong choices in clinical practice. Diagnostic accuracy refers to test characteristics like sensitivity, specificity, predictive values and likelihood ratios. If there is a test of which you are not sure about the diagnostic accuracy, you can put an X in the respective column. Please elaborate on your doubts in the column for comments.

3. Diagnostic thinking impact
The diagnostic thinking impact refers to the added value of a test. Does a positive or negative test result substantially enhance the probability that a certain disease is either present or absent? Will this test lead you to a diagnosis? If there is a test of which you are not sure about the diagnostic thinking impact, you can put an X in the respective column. Please elaborate on your doubts in the column for comments.

4. Therapeutic choice impact
What are the consequences of this test? Does a positive test imply a different approach compared to a negative test? Does the test result lead to a specific therapeutic choice? Can a patient be reassured with the test result? If there is a test of which you are not sure about the therapeutic choice impact, you can put an X in the respective column. Please elaborate on your doubts in the column for comments.

5. Patient outcome impact
Does the application of this test and the associated outcome lead to a more favorable patient outcome? If there is a test of which you are not sure about the patient outcome impact, you can put an X in the respective column. Please elaborate on your doubts in the column for comments.

6. Social impact
Social impact refers to the question whether a test is acceptable from a cost-effectiveness perspective. If a test shows great diagnostic accuracy values, it may still not be desirable because of its high costs. If there is a test of which you are not sure about the social impact, you can put an X in the respective column. Please elaborate on your doubts in the column for comments.

Missing tests
It is possible that you miss a specific diagnostic test. We would like to invite you to name this test and provide your argumentation for this specific test.

REFERENCES

1. Kovacs E, et al. Economic burden of vertigo: a systematic review. Health Econ Rev 2019;9(1):37. Economic burden of vertigo: a systematic review - PubMed (nih.gov)
2. World Population Ageing20 United Nations, Department of Economic and Social Affairs, Population Division: New York. World Population Ageing 2020
3. Bouma M, et al. Dutch Primary Care Guideline on Dizziness (in Dutch). Huisarts Wet 2017;60(7):348-356, Duizeligheid | NHG-Richtlijnen
4. Jones J, Hunter D. Consensus methods for medical and health services research. BMJ. 1995 Aug 5;311(7001):376-80. Consensus methods for medical and health services research - PubMed (nih.gov)
5. Meijer R, et al. The use of a modified Delphi procedure for the determination of 26 prognostic factors in the sub-acute stage of stroke. Int J Rehabil Res. 2003 Dec;26(4):265-70. The use of a modified Delphi procedure for the determination of 26 prognostic factors in the sub-acute stage of stroke - PubMed (nih.gov)
6. Powell C. The Delphi technique: myths and realities. J Adv Nurs. 2003 Feb;41(4):376-82. The Delphi technique: myths and realities - PubMed (nih.gov)
7. Tatsioni A, et al. Challenges in systematic reviews of diagnostic technologies. Ann Int Med 2005; 142: 1048-55. Challenges in systematic reviews of diagnostic technologies - PubMed (nih.gov)
