# Supplementary material for: Identifying tests to evaluate in a diagnostic accuracy study for patients with vertigo in general practice: a Delphi study
Source: BMC Prim Care. 2025 Aug 2;26:238. doi: 10.1186/s12875-025-02920-z (PMC12318412; doi:10.1186/s12875-025-02920-z)
Supplement: Supplementary file 2 — Supplementary Material 2. [file 12875_2025_2920_MOESM2_ESM.pdf]

APPENDIX

Delphi procedure VERDI study

DEPARTMENT OF GENERAL PRACTICE, AMSTERDAM UMC, THE NETHERLANDS

Drs. A.R. Leemeyer, MD, PhD student
Dr. VA van Vugt, GP, Postdoctoral Researcher and Epidemiologist
Dr. O.R. Maarsingh, GP, Associate Professor and Epidemiologist
Contact: a.leemeyer@amsterdamumc.nl

APPENDIX I

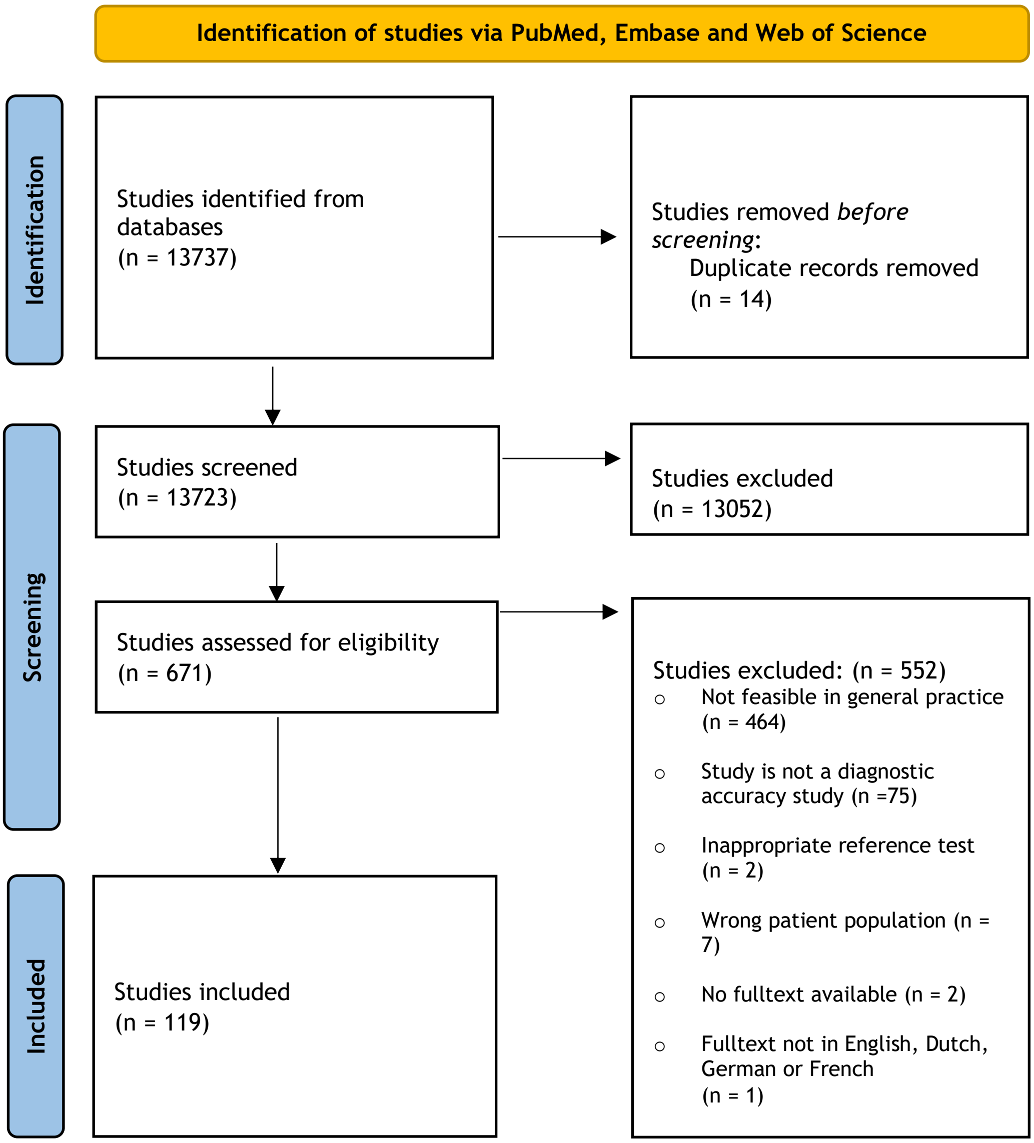

Figure: PRISMA flow diagram of systematic literature search to establish evidence on tests to diagnose causes of vertigo in general practice.

APPENDIX II

Diagnostic Test Page

I. PATIENT HISTORY 4
1. Present vertigo symptoms 4
2. Accompanying symptoms 4
3. Timing of symptoms 4
4. Triggers of symptoms 4
5. Medication and intoxication history 4
6. Past medical history 4
7. Cardiovascular risk factors 4

II. PHYSICAL EXAMINATION 5
8. Pulse and blood pressure 5
9. Otoscopy 6
10. Audiometry 6
11. Motor and sensory function (global neurology examination) 7
12. Nystagmus, general observation 7
13. Head-shaking nystagmus 8
14. HINTS (Head Impulse, Nystagmus, Test of Skew) 9
15. Sideways Stepping/Fukuda stepping/Unterberger Test 10
16. Dix-Hallpike 11
17. Head-roll/Supine-roll Test 12
18. STANDING (SponTAneous Nystagmus, Direction, head Impulse test, standiNG) 13
19. Gait Disorientation Test 14
20. Tandem Walking test 14
21. Bucket Test 15
22. Romberg Test 16
23. Single Leg Stance 17
24. Timed up & Go Test 17
25. Past Pointing Test 18
26. Five Times Sit to Stand Test 18
27. Pull Test 19
28. Frenzel glasses 19

III. ADDITIONAL TESTS 20
29. Laboratory tests 20

IV. QUESTIONNAIRES 21
30. PCI Score System 21
31. Essen Score System 22
32. ABCD2 23
33. TriAGe+ Score System 24
34. Questionnaires Benign Paroxysmal Positional Vertigo 25
35. Questionnaire Vestibular Migraine 26
36. Questionnaire Vestibular disorders 27

I. PATIENT HISTORY

1. Present vertigo symptoms
What kind of vertigo symptoms does the patient experience?

2. Accompanying symptoms
Does the patient experience other symptoms associated with vertigo symptoms (e.g. nausea, vomiting, tinnitus)?

3. Timing of symptoms
When does the patient experience his/her complaints of vertigo? Is it a constant feeling or are there attacks of vertigo? What is the duration of attacks (e.g. seconds, minutes, hours)? How often does it occur (daily, weekly, monthly)?

4. Triggers of symptoms
Does the patient describe activities/factors that provoke the vertigo (e.g. bending over, looking up, loud noises)?

5. Medication and intoxication history
Does the patient use medication? Does he/she smoke or use alcohol or drugs?

6. Past medical history
Does the patient have a medical history of cardiovascular diseases, internal diseases, ENT diseases, neurological diseases, musculoskeletal diseases, and/or visual diseases?

7. Cardiovascular risk factors
Does the patient have known cardiovascular risk factors (e.g. smoking, obesity, high blood pressure)?

II. PHYSICAL EXAMINATION

8. Pulse and blood pressure

Background:
Pulse measurement and blood pressure measurements can be performed to assess the hemodynamic condition of a patient. Hemodynamic abnormalities can be associated with vestibular symptoms.

Pulse measurement
Method:
Unilateral palpation of the arteria radialis in the sitting patient.
The frequency (per minute) and regularity (regular or irregular) of the pulse is determined. An irregular pulse should be measured over a longer period than a regular one and a slow rate should be timed for longer. As a guide, a regular rate is measured for 30 seconds and an irregular pulse or slow rate a full minute.

Explanatory references:
- Spodick, Am Heart J, 1992
- Birgnole et al, Eur Heart J, 2004

Blood pressure
Method:
The blood pressure must be measured while the patient is in a sitting position after at least five minutes rest. The measurement should be conducted with a manometer with an adequate cuff (the air holding compartment of the cuff must at least cover 80% of the diameter and 40% of the length of the upper arm). The cuff must be placed as high as the middle of patient’s sternum. The cuff is inflated until 20 mmHg above the moment the pulse disappears. During measurement, patient and physician are silent. The blood-pressure is measured with an accuracy of 2 mmHg per second.
The systolic blood-pressure is read when the first tone appears and the diastolic blood-pressure when the tone fully disappears (Korotkoff phase I and V).

Explanatory references:
- Thomas G et al, UpToDate, 2022

9. Otoscopy

Background:
Otoscopy can be used to diagnose inflammation/infection of the inner ear, perforation of the tympanic membrane, perilymphatic fistula or cholesteatoma. These diagnoses can be associated with vestibular symptoms.

Method:
Examination of the ear canal and eardrum with an otoscope.

Explanatory references:
- Limb, et al, UpToDate, 2022
- Bouma et al, Huisarts Wet, 2017

10. Audiometry

Background:
Audiometry can be used to assess a patient's hearing. Hearing abnormalities can be associated with vestibular symptoms.

Method:
The patient is sitting on a chair and cannot see the audiometric device. The examiner examines both ear canals. The audiometric evaluation contains 8 frequencies, with 120-250-500-1000-2000-4000-6000-8000Hz in ascending order. At each frequency, a 60 decibel (dB) tone is played. If the patient states that he/she can hear the tone, the examiner lowers the volume to 40 dB and repeats the question. If the patient state that he/she hears the tone, the physician keeps lowering the volume until the patient indicates that he/she can't hear the tone. The examination results in an audiogram that signifies if there is a loss of hearing.

Explanatory references:
- Weber P, et al. UpToDate, 2022

Systematic literature search (Appendix I):
- Di Giustino et al, Hearing, Balance and Communication, 2016

11. Motor and sensory function (global neurology examination)

Background:
The standard neurological clinical assessment is used to evaluate the nervous system. This exam is used to diagnose disorders affecting the brain, nerves and spinal cord that may cause vestibular symptoms.

Method:
The physician will perform a state-of-the-art global neurological clinical examination, focusing on motor function, sensory function and ataxia.

Explanatory references:

- Levin, Merck Manual, 2021
- Shahrokhi et al, StatPearls, 2022
- Hafiz et al, StatPearls, 2022

12. Nystagmus, general observation

Background:
A nystagmus is an involuntary eye movement, that is directly followed by a compensatory rapid eye movement in the contradictory direction. The occurrence of nystagmus can be associated with vestibular symptoms and problems within the vestibular system.

Method (general observation):
The patient is first observed for spontaneous nystagmus while looking straight ahead. Smooth pursuit is tested by asking the patient to follow the examiner's finger in all directions as the examiner moves the finger slowly up, down, left and right. To test if nystagmus occurs on lateral gaze, the patient is asked to look at the examiner's finger placed >60 degrees from midline, both left and right subsequently.

Explanatory references:

- Barton et al, UpToDate, 2019
- Bouma et al, Huisarts Wet, 2017
- Thurtell et al, Hand Clin Neuro: Chap 13, 2011

Systematic literature search (Appendix I):

- Califano et al, Acta Otorhinolaryngol Ital, 2017
- Chang et al, BMC Neurol, 2019

13. Head-shaking nystagmus (HSN)

Background:
The head-shaking nystagmus (HSN) is used to assess (unilateral) vestibular dysfunction. The HSN can be performed in different ways, depending on the clinician and his/her surroundings: 1. active versus passive, 2.darkened room yes/no, 3.use of Frenzel glasses yes/no, 4.shaking horizontally versus sinusoidally.

Method:
To perform the examination, the head of the seated patient is tilted forward 30°, then actively or passively rotated 45° toward each side 20 to 30 times at a rate of 1 to 2 Hz. The bedside HSN test is usually performed by having the patient wear Frenzel's or by direct visualization [Angeli et al, 2011].

Video material:
- Headshake Nystagmus - YouTube
- Head Shaking Nystagmus - YouTube

Systematic literature search (Appendix I):
- Angeli et al, Am j Otolaryngol, 2011
- Califano et al, Acta Otorhinolaryngol Ital, 2017
- Di Giustino et al, Hearing, Balance and Communication, 2016
- Gofrit et al, Ann Otol Rhinol Laryngol, 2017
- Kamal et al, Audiolog Med, 2011

14. HINTS

Background:
The HINTS exam consists of the Head Impulse Test (HIT), Nystagmus examination and Test of Skew. The HIT is also known as the Head Thrust Test (HTT).
The HINTS is a clinical bedside test that is used to differentiate peripheral vestibular disease from stroke in patients with an acute vestibular syndrome (AVS).
Two variations of the HINTS are described: the “standard” HINTS and the HINTS plus.

Method HINTS:
1. Head Impulse Test (HIT)/Head Thrust Test (HTT):
The physician, facing and holding the patient’s head at arm’s length, performs a passive and unpredictable head rotational movement in a high acceleration ~ 20 degree to either side having the patient’s eyes fixed on the examiner’s nose [Maranhão et al, 2016].

Positive test (classified as pathologic): When the eye is no longer compensating for the head movement and there is a corrective saccade observed”.

2. Nystagmus examination
Acute Vestibular Syndrome (AVS) should generally be associated with a characteristic, dominantly horizontal nystagmus that beats only in one direction and increases in intensity when the patient looks in the direction of the nystagmus fast phase. Vertical or torsional nystagmus in this clinical context is a clear sign of central pathology, but most strokes presenting an AVS picture have nystagmus with a predominantly horizontal vector that mimics acute peripheral vestibulopathy. What sometimes distinguishes the nystagmus typical of central AVS from APV is a change in direction on eccentric gaze [Kattah et al, 2009].

3. Test of Skew
Skew deviation is vertical ocular misalignment that results from a right–left imbalance of vestibular tone, particularly otolithic inputs, to the oculomotor system. Skew is generally detected by alternate cover testing, i.e. covering/uncovering each eye in turn while observing eye movements [Kattah et al, 2009].

Positive test: Vertical ocular misalignment (skew deviation).

Video:
HINTS TO DIAGNOSE STROKE - YouTube
The HINTS exam - YouTube

Method HINTS Plus:
The HINTS Plus exam consists of the HINTS exam, combined with the assessment of new hearing loss. Hearing loss is judged to be present if bedside examination (finger rubbing) detects a clear right-left asymmetry and the patient confirms the deficit to be new [Newman-Toker et al, 2013].

Explanatory references:
- Kattah et al, Stroke, 2009
- Nham et al, J Neurol, 2022
- Kerber, Neurology, 2015

Systematic literature search (Appendix I):
- Newman-Toker et al, Acad Emerg Med, 2013
- Carmona et al, Front Neurol, 2016

- Choi et al, J Neurol, 2018
- Dmitriew et al, Acad Emerg Med, 2021
- Gerlier et al, Acad Emerg Med, 2021
- Korda et al, J Neurol, 2022
- Tehrani et al, Neurology, 2014
- Sankalia et al, Neurol India, 2021
- Wang et al, Acad Emerg Med, 2021

15. Sideways Stepping Test / Fukuda / Unterberger

Background:
The stepping test (known as Sideways Stepping test, Fukuda stepping test or Unterberger test) is a clinical test that is used to assess unilateral vestibular hypofunction.

Method:
1. The patient is asked to stand with their feet together and their hands by their sides. The patient is then asked to close his/her eyes or is blindfolded [Al Saif et al, 2014].

Positive test: If the patient leans voluntary to one side and hip sway.
Sometimes, the patient lifts a hand to compensate. In that case, the patient is asked to take two steps sideways and stop, first with the eyes open and then with the eyes closed or blindfolded. The test is considered positive if there is any involuntary swaying and/or any steps taken to compensate.

2. The patient is then asked to jog on the spot for 30 seconds with the eyes closed or blindfolded. There should be no deviation [Al Saif et al, 2014].

Positive test: The test is positive if there is any involuntary drifting to one side or, in some cases, forward or backward.

Video:
Fukuda Step Test (Vestibular Examination) - YouTube

Explanatory references:
- Hornibrook, Int J Otolaryngol 2012
- Honaker et al, J Am Acad Audiol, 2009
- Fukuda, Acata Otolaryngol, 1959

Systematic literature search (Appendix I):
- Al Sait et al, J Phys Ther Sci , 2014
- Cohen et al, Otolaryngol Head Neck Surg, 2014
- Honaker et al, J Am Acad Audiol, 2012
- Kamal et al, Audiological Medicine, 2011
- Maranhão et al, Arq Neuropsiquitr, 2016

16. Dix-Hallpike

Background:
The Dix-Hallpike maneuver is a clinical test for benign paroxysmal positional vertigo (BPPV) of the posterior semicircular canal. Several methods of performing the Dix-Hallpike are described: the standard Dix-Hallpike, loaded Dix-Hallpike and smartphone Dix-Hallpike.

Method:
Ling et al 2021 described the standard Dix-Hallpike as follows: “The patient's head was turned with the nose pointing 45° toward the side to be examined, and then the patient was moved quickly into a supine position with the head hanging about 20° over the end of the examination table. This position was maintained for at least 1 minute or until the induced nystagmus resolved. Then, while the patient 's head was kept turned 45° toward the side being examined, the patient was brought back to the sitting-up position.”

Andera et al 2020 described the loaded Dix-Hallpike as follows: “Head rotated 45 degrees to the affected side and flexed forward 30 degrees in the plane of the posterior canal for 30 seconds. Subsequently, the patients were reclined to their back with head extended to 20 degrees (same as the standard Dix-Hallpike).”

Shah et al 2019 described the smartphone Dix-Hallpike as follows: “The physician performed a DHT on the patient in a standard stretcher and used a smartphone to record the patients eye movements during the test. The recording was later remotely reviewed by two board-certified neuro-otologists for BPPV screening.”

Positive test: Experiencing vertigo during the maneuver. The observation of typical torsional, upbeating nystagmus confirms the diagnosis of BPPV.

Video:
Dix Hallpike Test | Posterior BPPV - YouTube

Explanatory references:
- Bouma et al, Huisarts Wet, 2017

Systematic literature search (Appendix I):
- Andera et al, J Int Adv Otol, 2020
- Evren et al, Braz J Otorhinolaryngol, 2017
- Shah et al, Laryngoscope, 2019
- Zainun et al, Int Medical J, 20133
- Jeon, J Vestib Res, 2019
- Ling et al, J Clin Neurol 2021
- Noda et al, Int J Gen Med, 2011
- Burston, J Clin Neurosci, 2012
- Kamal et al, Audiological Medicine, 2011

17. Head-roll/Supine-roll test

Background:
The Head-roll test is a clinical test for benign paroxysmal positional vertigo (BPPV) of the lateral (horizontal) semicircular canal.

Method:
The supine roll test is performed by initially positioning the patient supine with the head in neutral position, followed by quickly rotating the head 90 degrees to one side with the clinician observing the patient's eyes for nystagmus. After the nystagmus subsides (or if no nystagmus is elicited), the head is then returned to the straight face up supine position. After any additional elicited nystagmus has subsided, the head is then quickly turned 90° to the opposite side, and the eyes are once again observed for nystagmus [Bhattacharyya et al, 2017].

Positive test: Experiencing vertigo during the maneuver. The observation of nystagmus: initially the nystagmus will beat towards the affected ear, geotrophic (towards the floor) or apogeotropic (towards the ceiling).

Video:
Supine Head Roll Test | Lateral BPPV - YouTube

Explanatory references:
- Bouma et al, Huisarts Wet, 2017
- Asprella-Libonati, Acta Otorhinolaryngol Ital, 2008
- Bhattacharyya et al, Otolaryngol Head Neck Surg, 2017

Systematic literature search (Appendix I):
- Martellucci et al, Front Neurol, 2020

18. STANDING algorithm

(SponTAneous Nystagmus, Direction, head Impulse test, standiNG)

Background:
The STANDING algorithm is a clinical tool to differentiate peripheral vestibular disorders and central vestibular disorders.

Method [Vanni et al, 2014]:
(1) The patient is in supine position. If there is a spontaneous nystagmus present in the main gaze positions, the presence of a positional nystagmus is assessed by the Supine-roll test first and then by the Dix-Hallpike test. If a nystagmus is present, it's considered typical of BPPV.
(2) When spontaneous nystagmus already is present in supine position and persistent, the direction is examined: multidirectional nystagmus, such as bidirectional gaze-evoked nystagmus or torsional nystagmus are considered signs of central vertigo.
(3) When the nystagmus is unidirectional, a head impulse test is performed (HIT). For test description see page 9 of this appendix (14. HINTS).
(4) Patients showing neither spontaneous nor positional nystagmus are invited to stand and the gait is evaluated. When there is an inability to maintain an upright stance without assistance, they are suspected to have central disease.

Systematic literature search (Appendix I):
- Vanni et al, Emerg Med Australes, 2015
- Vanni et al, Acta Otorhinolaryngol Ital, 2014
- Vanni et al, Front Neurol, 2017
- Gerlier et al, Acad Emerg Med, 2021

19. Gait Disorientation Test

Background:
The Gait Disorientation (GDT) is developed from specific items from the Functional Gait Assessment (FGA). The FGA is an instrument to assess postural stability during walking and assesses an individual's ability to perform multiple motor tasks while walking. The GDT differentiates healthy patients from patients with vestibular impairment.

Method:
The GDT is constructed by comparing task 8 (walking with eyes closed) to task 1 (walking with eyes open) of the FGA. The GDT time is calculated by subtracting the time needed to complete task 1 from the time needed to complete task 8 [Grove et al, 2021].

Positive test: GDT time of ≥4.5 seconds.

Video:
Functional Gait Assessment
N.B. Task 1= Gait Level Surface (2:01), Task 8 = Gait With Eyes closed (6:03)

Explanatory references:
- Wrisley et al, Phys ther, 2004

Systematic literature search (Appendix I):
- Grove et al, Arch Phys Med Rehabil, 2021
- Chen et al, J Neurol, 2011

20. Tandem Walking Test

Background:
The Tandem walking Test is a clinical test used to screen patients for neurologic and vestibular disorders.

Method:
Mahadi et al, 2020: "The patient is asked to walk heel to toe on a straight line for 20 steps at his own pace. The ability to see the seam in the linoleum floor was checked. Counting the steps commenced once the participant started placing one foot before the other and stopped once a foot touched the floor before proper placement, the heel was not touching the toes, the foot was not placed on the line or the 20 steps limit was reached. The last unsuccessful step was not incorporated in the score."

Positive test: If the 20 steps limit could not be reached.

Video:
Assessment - Gait - Tandem Gait - YouTube

Systematic literature search (Appendix I):
- Salah et al, Otol Neurotol, 2020
- Cohen et al, J Vestiv Res, 2012
- Cohen et al, Otol Neurotol, 2019
- Cohen et al, Otolaryngol Head Neck Surg, 2014
- Cohen et al, Laryngoscope, 2018
- Longridge et al, Otol neurotol, 2010

21. Bucket Test

Background:
The Bucket Test (BT) is a clinical test that measures the difference between the patient's subjective perception of the visual vertical (SVV) and the true vertical. This can be helpful to distinguish central from peripheral acute vestibular syndrome.
Several methods of performing the Bucket test are described: the standard Bucket Test and the Bucket Test Smartphone.

Method Standard BT:
The patient sits upright and looks into a translucent plastic bucket. On the bottom, inside the bucket, there is a line of stars that glow in the dark. On the bottom, outside the bucket, there is a tool can measure degrees. The patient is given the bucket with the stars initially oriented horizontally, asked to place his/her face inside the bucket and to turn the bucket around in order to vertically align the stars on the interior surface of the bucket. The difference in degrees between the patient's SVV and the true vertical alignment (SVV tilt) is documented for each attempt, to left or right of the vertical 0. The test is repeated 3 times [Shaban et al, 2021]

Method BT Smartphone:
Instead of the bucket a smartphone (with a straight red line visible) is anchored to a rotating disk, fixed on the wall. The test is carried out in the dark, so the patient only sees a red line in a black background – in order to avoid the use of visual cues in the testing environment. Then, the physician slowly rotates the smartphone until the patient notifies verticality [Riera-Tur et al, 2022].

Positive test: If the patient's SVV is >2° different than the true vertical this is considered a positive test. If the patient misaligns the bucket on 2 out of 3 attempts and the deviation is consistently towards one side this is also considered a positive test.

Video:
Bucket test - YouTube

Explanatory references:
- Zwergal et al, Neurology, 2009

Systematic literature search (Appendix I):
- Riera-Tur et al, J Vestib Res, 2022
- Shaban et al, J Emerg Med, 2021
- Sun et al, Acta Otolaryngol, 2014
- Wengier et al, Oto Neurotol, 2021
- Chang et al, BMC Neurol, 2019
- Cohen et al, Acta Otolaryngol, 2012

22. Romberg Test

Background:
The Romberg test is a clinical test used to assess the dorsal columns of the spinal cord and the general balance of the patient. Vestibular dysfunction could be accompanied by balance problems. Several methods of performing the Romberg Test are described, i.e. the standard Romberg test, the Modified Romberg test using Foam pad (MRuFP) and the Tandem Romberg.

Method:
Salah et al (2020) described the standard Romberg Test as follows: “The patient is instructed to stand with their eyes closed, arms in specific position, feet together for a maximum of 30 seconds.”

Hong et al (2015) described the MRuFP as follows: “The patient is asked to stand erect with their feet 10 cm apart, and arms folded with the hands cupping the elbows on a firm surface and on a polyurethane foam pad (50 × 50 × 6 cm). The patient is then asked to try to keep his/her body as stable as possible under four conditions: on a firm surface with (1) eyes open and (2) eyes closed, and on the foam pad with (3) eyes open and (4) eyes closed.”

Longridge et al (2010) described the Tandem Romberg Test as follows: “The patient is asked to stand in a tandem position (heel touching toe) for 20 seconds.”

Positive test for the standard Romberg Test and the MRuFP:
Patients who cannot maintain balance for 30 sec are deemed to have failed the test under that condition; failure to maintain balance is defined as: (1) the patient’s feet moved from the initial position, (2) the patient’s arms moved off the waist, or (3) the patient’s eyes opened during an EC test.

Positive test for the Tandem Romberg Test: If the patient has to take a step to the side.

Video:
Romberg Test - (Romberg's Test) - YouTube
Tandem Romberg Test - YouTube

Systematic literature search (Appendix I):

- Salah et al, Otol Neurotol, 2020
- Zamyslowska-Szmytke et al, Int J Occup Med Environ Health, 2015
- Cohen et al, Otol Neurotol, 2019
- Gofrit et al, Ann Otol Rhinol Laryngol, 2017
- Hong et al, Eur Arch Otorhinolaryngol, 2015
- Longride et al, Otol Neurotol, 2010

23. Single Leg Stance Test

Background:
The Single Leg Stance Test is a clinical test that assesses static postural and balance control.

Method:
The patient is barefoot and asked to stand on one leg while the arms are free to move. They can choose which leg they want to stand on and they can alternate between legs in between trials. The test will be performed with the eyes open and closed. Timing starts when the patient closes his/her eyes while standing on one leg or when raising one foot of the ground. The timing stops when the patient repositions the weight-bearing foot, touches the floor with the suspended foot, uses the suspended foot for support on the weight-bearing foot, requires support by the investigator, opens his eyes, or reaches the maximum time of 30 seconds. [Salah et al, 2020]

Positive test: If the patient is unable to perform the test properly which leads to ending the time before the 30 second limit.

Video:
- What Single Leg Stance Assessment can tell you - YouTube

Systematic literature search (Appendix I):
- Salah et al, Otol Neurotol, 2020

24. Timed Up & Go Test

Background:
The Timed Up & Go Test is a clinical test to asses a patients mobility and balance.

Method:
The patient is asked to sit on a standard (arm) chair with his/her back against the chair and feet flat on the floor. The patient is instructed — on the word "start", after the warning "ready" — to rise and to walk as fast as possible to a mark on the floor 3m away, turn around, walk back to the chair, and sit down again. Timing commenced on the word "start" and ceased once the patients back touched the back of the chair. The patient performs the test three times with his/her preferred turn (first choice) and then three times with a turn to the other side. The fastest time is considered for analysis [Mahadi et al, 2020].

Positive test: If the patient needs more than 20 seconds to complete the task.

Video:
The Timed Up and Go Test (TUG) | Fall Risk Assessment - YouTube

Systematic literature search (Appendix I):
- Maranhão et al, Arq Neuropsiquitr, 2016
- Salah et al, Otol Neurotol, 2020

25. Past Pointing Test

Background:
The Past Pointing Test is a clinical test that evaluates the upper extremity balance (e.g. the functional integrity of the vestibular apparatus and the cerebellum).

Method:
The patient is instructed to extend the arms and place the index finger of one hand on the index finger of the examiner. The eyes are then closed, the arm raised above the head, then quickly returned to the perceived starting position. The procedure is repeated five times [Maranhão et al 2016].

Positive test: The patient drifts away from the target towards the compromised side.

Video:
Past Pointing Test - YouTube

Systematic literature search (Appendix I):
- Maranhão et al, Arq Neuropsiquitr, 2016

26. Five Times Sit to Stand

Background:
The Five Times Sit to Stand is a clinical tool to measure functional lower extremity strength and the ability to make transfers.

Method:
The patient is seated in a chair with arms crossed under chest and feet fitted on the ground. Upon request, the patient stands up and sits down for 5 times. Time is measured with a stopwatch [Maranhão et al 2016].

Positive test: More than 10 seconds to complete the task.

Video:
Five Time Sit to Stand Test (FTSST) - YouTube

Explanatory references:
- Guralnik et al, J Gerontol, 1994

Systematic literature search (Appendix I):
- Maranhão et al, Arq Neuropsiquitr, 2016

27. Pull Test

Background:
The Pull test is a clinical test that assesses the postural stability.

Method:
The patient stands with the feet slightly spread apart and is pulled backward by the shoulders (one or two steps backwards allowed) [Maranhão et al 2016].

Positive Test: If the patient has to take 2-3 steps te maintain balance or fall backward like a log.

Systematic literature search (Appendix I):
- Maranhão et al, Arq Neuropsiquitr, 2016

28. Frenzel glasses

Background:
A characteristic of the peripheral vestibular nystagmus is that it can be suppressed by visual fixation. Frenzel goggles can reduce or remove visual fixation and help to assess nystagmus.

Method:
The Frenzel goggles are a pair of magnifying glasses worn by the patient [Yeolekar et al, 2019].

Explanatory references:
- Nystagmus goggles: how to use them, what you find and what it means

Systematic literature search (Appendix I):
- Yeolekar et al, Clin Med insights Ear Nose Throat, 2019

III. ADDITIONAL TESTS

29. Laboratory Testing

Background:
Several studies describe possible associations between laboratory tests and vestibular disorders.

BPPV could be associated with an increased lipid profile (Celibilek; Evrin), albumin (Celikbilek), plateletcrit (Evrin), LDL/HDL ratio, neutrophil to lymphocyte ratio, triglyceride and serum uric acid. However, an association with BPPV was also found with decreased values of serum uric acid (Celikbilek), HbA1c, albumin, and creatinine (Yuan).

Systematic literature search (Appendix I):
- Celikbilek et al, Eur J Neurol, 2014
- Evrin et al, Eurasion J Emerg Med, 2019
- Lan et al, Ann Palliat Med, 2021
- Yuan et al, Med Sci Monit, 2017

Central vertigo could be associated with an increased white blood count (Hong; Evrin), hemoglobin (Hong), neutrophil to lymphocyte ratio (Lan; Lee; Zhang), neutrophil count, triglyceride (Lan), LDL/HDL ratio (Lan), fibrinogen (Wang), and creatinine (Hong). There could also be an association with an increased level of biomarkers: miR-125a-5p, miR-125b-5, and miR-433-5p (Kijpaisalratana), S100 calcium-binding protein B (Kartal; Mozafari; Purrucker; Sohn), neuron-specific enolase (Mozafari; Sohn), matrix metalloproteinase 9 (Purrucker), ischemia-modified albumin (IMA) levels (Sener) and thiol/disulfide homeostasis (TDH) parameters (Sener).

Systematic literature search (Appendix I):
- Hong et al, Front Neurol, 2021
- Lee et al, J Emerg Med, 2018
- Zhang et al, Clin Lab, 2019
- Kijpaisalratana et al, BMC Neurol, 2020
- Kartal et al, Acad Emerg Med, 2014
- Mozafari et al, Arch Acad Emerg Med, 2019
- Sohn et al, Frond Med (Lausanne), 2020
- Şener et al, Annals of Clinical and Analytica Medicine, 2021
- Purrucker et al, Euro Neurol, 2014

Vestibular migraine could be associated with a decreased level of magnesium and hemoglobin and an increase in platelet count and folic acid (Zhou).

Systematic literature search (Appendix I):
- Zhou et al, Neuropsychiatr Dis Treat, 2020

IV. QUESTIONNAIRES

30. PCI Score System

Background:

The PCI score system is a clinical risk score that is used to differentiate patients with vertigo and posterior circulation ischemia (PCI) from other dizzy patients.

Method:

Risk factors	Scores
High blood pressure	1
Diabetes mellitus	1
Ischemic stroke	1
Rotating and rocking	−1
Difficulty in speech	5
Tinnitus	−5
Limb sensory deficit	5
Gait ataxia	1
Limb ataxia	5

Positive test: The higher the score, the greater the probability of PCI.

Systematic literature search (Appendix I):

- Chen et al, J Stroke Cerebrovasc Dis, 2018

31. Essen Score System

Background:
The Essen risk score is a clinical score that is used to predict the 1-year risk of recurrent ischemic stroke after ischemic stroke.

Methods:

Variables of the Essen Score	Points
Age, year	
<65	0
65-75	1
>75	2
History of hypertension	1
History of diabetes mellitus	1
History of peripheral artery disease	1
History of previous myocardial infarction	
History of other cardiovascular disease (except myocardial infarction and atrial fibrillation)	1
Current smoking	1
Additional transient ischemic attack or ischemic stroke in addition to the qualifying event (ie, previous TIA/ischemic stroke)	1

Positive test: a higher risk score indicates a large chance of recurrent ischemic stroke

Explanatory references:
- Boulanger et al, Stroke, 2019

Systematic literature search (Appendix I):
- Chen et al, J Stroke Cerebrovasc Dis, 2018

32. ABCD^2 Score System

Background:
The ABCD^2 score helps to predict the risk of stroke after having a transient ischemic attack.

Methods:

Risk factor	Points
Age≧60	1
Blood pressure: Systolic≧140 or diastolic≧90	1
Clinical features: Unilateral weakness	2
Speech disturbance	1
Duration: ≧60 minutes	2
1-59 minutes	1
Diabetes mellitus	1

Positive test: ABCD^2 scores ≧4 are defined as having a high risk of a future cerebrovascular event.

Systematic literature search (Appendix I):

- Chang et al, BMC Neurol, 2019
- Chen et al, J Stroke Cerebrovasc Dis, 2018
- Gerlier et al, Acad Emerg Med, 2021
- Kerber et al, Neurology, 2015
- Kuroda et al, J Stroke, Cerebrovasc Dis, 2017
- Navi et al, Stroke, 2012
- Newman-Toker et al, Acad Emerg Med, 2013
- Wang et al, Acad Emerg Med, 2021
- Chang et al, BMC Neurol, 2019

33. TriAGe+ Score System

Background:
The TriAGe+ score is used to identify stroke in patients with vestibular symptoms presenting to the emergency department.

Methods:

Initials	Variables	Points
Tri	No triggers	2
A	Atrial fibrillation	2
Ge	Gender (=male)	1
B	Blood pressure (≥140/90) "B" of ABCD2	2
B	Brainstem or cerebellar dysfunction*	1
C	Clinical features (focal weakness or speech impairment)	4
	"C" of ABCD2	
D	Dizziness (=not vertigo)	3
H	No history of vertigo or dizziness or labyrinth or vestibular disease	2

*

Brainstem or cerebellar dysfunction: skew deviation, diplopia, disturbance of ocular movement, unilateral disturbance of facial sensation, dysphagia, hiccup, dysmetria, and truncal ataxia.

Positive test: A higher TriAGe+ score indicates a higher risk of stroke as a cause of vestibular symptoms.

Systematic literature search (Appendix I):

- Kuroda et al, J Stroke, Cerebrovasc Dis, 2017

34. Questionnaires Benign Paroxysmal Positional Vertigo

Background:
Questionnaire used to identify patients with Benign Paroxysmal Positional Vertigo (BPPV).

Methods:
Several questionnaires for diagnosing BPPV have been described:

Chen et al 2016, questionnaire *(part of the total Dizziness Handicap Inventory)*:

- Five sub-scale items
  - Does looking up increase your problem?
  - Does bending over increase your problem?
  - Do quick movements of your head increase your problem?
  - Because of your problem, do you have difficulty lying down or getting out of bed?
  - Does rolling over in bed increase your problem?
- Two sub-scale items
  - Because of your problem, do you have difficulty lying down or getting out of bed?
  - Does rolling over in bed increase your problem?

Each question provides a choice of 3 responses: yes (4 points), sometimes (2 points) or no (0 point).

Positive Test: The 5-item questionnaire has a cutoff value of 12 points and the 2-item questionnaire has a cutoff value of 6; both may indicate BPPV.

Systematic literature search (Appendix I):

- Chen et al, Neurol Sci, 2016

Kim et al, 2020, questionnaire:

1. Do you have spinning or a whirling sensation of the surroundings or yourself?
2. Do you feel dizzy mostly when your head is moved?
3. Does the dizziness last <3min?

Positive result: If patient say “no” to any of these questions, they are presumed to have a disorder other than BPPV.
But if patients answer “yes” to all 3 questions (most likely BPPV), they proceed to the following 3 questions:

4. Which positional change makes you feel more dizzy? *(to determine the affected canal)*
   a. Lying down or getting out of bed?
   b. Turning your head (or body) while lying down?
5. Which makes you more dizzy? *(to determine the involved side)*
   a. Turning your head to the right?
   b. Turning your head to the left?
6. How long does the dizziness induced by head turning last? *(to determine the subtype)*
   a. <1 min?
   b. >1 min?

Systematic literature search (Appendix I):

- Kim et al, Neurology, 2020

Lapenna et al, 2016, questionnaire;

1. Sudden onset of rotatory vertigo or rollover feeling	Score = 1.2
2. Relapsing crisis each lasting from seconds to minutes	Score = 0.7
3. Crisis triggered by movement of the head, especially in the supine position	Score = 5.3
4. Relative comfort with the head still	Score = 2
5. Presence of nausea and/or vomiting	Score = 0.2
6. Absence of other audiological symptoms or cephalagia	Score = 0.6
	Maximum = 10

Positive test: A score of >2 may indicate BPPV.

Systematic literature search (Appendix I):

- Lapenna et al, Aging clin Exp Res, 2016

35. Questionnaire Vestibular Migraine

Background:

Questionnaire used to identify patients with vestibular migraine.

Celebisoy et al, 2016, questionnaire:

1. What is your main complaint?
    a. Vertigo, unsteadiness
    b. Light-headedness, near-faint feelings, loss of consciousness
2. How is the course of complaints
    a. Go with recurrent attacks
    b. Continuous
3. If you experience recurrent attacks what can you say about the duration in most of the episodes?
    a. Minutes to days
    b. Less than a minute or longer than a week
4. Are your complaints provoked just by positional changes such as turning in bed or looking up?
    a. Yes
    b. No
5. Do you experience accompanying hearing problems such as hearing loss, tinnitus, aural pressure during your complaints?
    a. Yes
    b. No
6. Do you experience headache during your complaints in around half of the attacks
    a. Yes
    b. No
7. Do the headache attacks last more than four hours without treatment?
    a. Yes
    b. No
8. Do you headaches display at least two of the below characteristics?
    - Unilateral location
    - Pulsating quality
    - Moderate or severe intensity
    - Aggravation by physical activity
    a. Yes
    b. No

Positive test:
If the patient has selected option B in question 4 and 5 and option A in all the other questions, than the diagnosis would be most likely vestibular migraine.

Systematic literature search (Appendix I):
- Celebisoy et al, Neurologist, 2016

36. Questionnaire Vestibular disorders

Stewart et al, 2015, questionnaire:
Background:
Patient-reported outcome measure for application in the acute hospital setting to screen for non-emergent vestibular disorders.

Method:
1. Do you have a feeling that things are spinning or moving around?
2. Does bending over or looking up at the sky make you feel dizzy?
3. Does lying down and/or turning over in bed make you feel dizzy?
4. Does moving your head quickly from side to side make you feel dizzy?

Score: yes = 2, sometimes = 1, no = 0.

Positive test:
The 4-item vestibular screening tool is scored out of 8, with a cutoff score ≥4 indicative of a vestibular disorder.

Systematic literature search (Appendix I):
- Stewart et al, Arch Phys Med Rehavil, 2015

Bayer et al, 2010, questionnaire:
Background:
Screening tool for vertigo in clinical practice: Benign Paroxysmal Positional Vertigo (BPPV), Menière's Disease (MD), Vestibular Migraine (VM) and Phobic Postural Vertigo (PPV).

(Method: see next page)

Method:

Questions used to build the diagnostic indices

How does your vertigo occur?	in attacks	as persistent vertigo	as persistent vertigo with attacks	
What kind of vertigo do you have?	rotational vertigo	unsteadiness	feeling of being in a lift	lightheaded ness
How do you perceive the environment during vertigo?	like on a roundabout	like on a boat	very blurred	
How often do you have defective hearing?	never	occasionally	frequently	always
How often do you have ear noises?	never	occasionally	frequently	always
How often do you have sweating/nausea/vomiting?	never	occasionally	frequently	always
How often do you have drop seizures?	never	occasionally	frequently	always

Positive test: The tool is used to calculate a score per target condition. Each target condition has his own cutoff value.

Systematic literature search (Appendix I):

- Bayer et al, BMC Neurol, 2010
