# Supplementary material for: Identifying tests to evaluate in a diagnostic accuracy study for patients with vertigo in general practice: a Delphi study
Source: BMC Prim Care. 2025 Aug 2;26:238. doi: 10.1186/s12875-025-02920-z (PMC12318412; doi:10.1186/s12875-025-02920-z)
Supplement: Supplementary file 3 — Supplementary Material 3. [file 12875_2025_2920_MOESM3_ESM.pdf]

Systematic Review VERDI-study

Search strategy

To identify the relevant publications we conducted systematic searches in the bibliographic databases PubMed, Embase.com and Web of Science (Core Collection) from inception to March 11, 2022, in collaboration with a medical information specialist. The following terms were used (including synonyms and closely related words) as index terms or free-text words: "Vertigo", "Dizziness", "Diagnostic accuracy", "Adults".

Duplicate articles were excluded using Endnote X21.0.1 (Clarivate^{tm}), following the Amsterdam Efficient Deduplication (AED)-method (Otten et al., 2019) and DedupEndNote (Lobbestael, 2023).

Search results

The literature search generated a total of 34375 references: 10464 in PubMed, 11784 in Embase.com and 12127 in Web of Science. After removing duplicates of references that were selected from more than one database, 13737 references remained. The flow chart of the search and selection process is presented in figure 1.

PubMed Session Results (11 Mar 2022)

Search	Query
#7	**#6 AND (("2010/01/01"[EDAT] : "3000/01/01"[EDAT]) OR ("2010/01/01"[PDAT] : "3000/01/01"[PDAT]))**
#6	**#5 AND ("Sensitivity and specificity"[MeSH] OR predict*[tw] OR diagnos*[tw] OR accura*[tw])**
#5	**#4 NOT ("address"[Publication Type] OR "autobiography"[Publication Type] OR "bibliography"[Publication Type] OR "biography"[Publication Type] OR "pubmed books"[Filter] OR "case reports"[Publication Type] OR "clinical conference"[Publication Type] OR "clinical trial, phase i"[Publication Type] OR "clinical trial, phase ii"[Publication Type] OR "clinical trial, phase iii"[Publication Type] OR "clinical trial, phase iv"[Publication Type] OR "technical report"[Publication Type] OR "clinical trial, veterinary"[Publication Type] OR "comment"[Publication Type] OR "hascommenton"[All Fields] OR "congress"[Publication Type] OR "consensus development conference"[Publication Type] OR "consensus development conference, nih"[Publication Type] OR "dictionary"[Publication Type] OR "directory"[Publication Type] OR "duplicate publication"[Publication Type] OR "editorial"[Publication Type] OR "electronic supplementary materials"[Publication Type] OR "festschrift"[Publication Type] OR "government publication"[Publication Type] OR "guideline"[Publication Type] OR "historical article"[Publication Type] OR "interactive tutorial"[Publication Type] OR "interview"[Publication Type] OR "introductory journal article"[Publication Type] OR "lecture"[Publication Type] OR "legal case"[Publication Type] OR "legislation"[Publication Type] OR "letter"[Publication Type] OR "meta analysis"[Publication Type] OR "news"[Publication Type] OR "newspaper article"[Publication Type] OR "observational study, veterinary"[Publication Type] OR "patient education handout"[Publication Type] OR "periodical index"[Publication Type] OR "personal narrative"[Publication Type] OR "portrait"[Publication Type] OR "practice guideline"[Publication Type] OR "research support, american recovery and reinvestment act"[Publication Type] OR "review"[Publication Type] OR "scientific integrity review"[Publication Type] OR "systematic review"[Filter] OR "twin study"[Publication Type] OR "webcast"[Publication Type])**
#4	**#3 NOT ("Animals"[Mesh] NOT "Humans"[Mesh])**
#3	**#2 NOT (("Adolescent"[Mesh] OR "Child"[Mesh] OR "Infant"[Mesh] OR adolescen*[tiab] OR child*[tiab] OR schoolchild*[tiab] OR infant*[tiab] OR girl*[tiab] OR boy[tiab] OR boys[tiab] OR boyhood[tiab] OR teen[tiab] OR teens[tiab] OR teenager*[tiab] OR youth*[tiab] OR pediatr*[tiab] OR paediatr*[tiab] OR puber*[tiab]) NOT ("Adult"[Mesh] OR adult*[tiab] OR man[tiab] OR men[tiab] OR woman[tiab] OR women[tiab]))**
#2	**#1 AND (dutch[la] OR english[la] OR french[la] OR german[la])**
#1	**"Vertigo"[Mesh] OR "Dizziness"[Mesh] OR vertig*[tiab] OR dizz*[tiab] OR vestibul*[tiab]**

Embase.com Session Results (11 Mar 2022)

Search	Query
#7	**#6 AND [2010-3000]/py**
#6	**#5 AND ('sensitivity and specificity'/exp OR predict*:ab,ti,kw,de OR diagnos*:ab,ti,kw,de OR accura*:ab,ti,kw,de)**
#5	**#4 NOT ([conference abstract]/lim OR [conference paper]/lim OR [conference review]/lim OR [data papers]/lim OR [editorial]/lim OR [erratum]/lim OR [letter]/lim OR [note]/lim OR [review]/lim OR [short survey]/lim)**
#4	**#3 NOT ([animals]/lim NOT [humans]/lim)**
#3	**#2 NOT (('adolescent'/exp OR 'child'/exp OR adolescent*:ab,ti,kw OR child*:ab,ti,kw OR schoolchild*:ab,ti,kw OR infant*:ab,ti,kw OR girl*:ab,ti,kw OR boy*:ab,ti,kw OR teen:ab,ti,kw OR teens:ab,ti,kw OR teenager*:ab,ti,kw OR youth*:ab,ti,kw OR pediatr*:ab,ti,kw OR paediatr*:ab,ti,kw OR puber*:ab,ti,kw) NOT ('adult'/exp OR 'aged'/exp OR 'middle aged'/exp OR adult*:ab,ti,kw OR man:ab,ti,kw OR men:ab,ti,kw OR woman:ab,ti,kw OR women:ab,ti,kw))**
#2	**#1 AND (dutch:la OR english:la OR french:la OR german:la)**
#1	**'vertigo'/exp/mj OR 'dizziness'/exp/mj OR vertig*:ab,ti,kw OR dizz*:ab,ti,kw OR vestibul*:ab,ti,kw**

Web of Science (Core Collection) Session Results (11 Mar 2022)

Search	Query
#6	**#5 AND DOP=(2010-01-01/2022-12-31)**
#5	**#4 AND TS=(predict* OR diagnos* OR accura*)**
#4	**#3 AND (Review Articles or Meeting Abstracts or Proceedings Papers or Editorial Materials or Letters or Notes or Book Reviews or Book Chapters or Reprints or Poetry or News Items or Record Reviews or Discussions or Film Reviews or Dance Performance Reviews or Art Exhibit Reviews or Data Papers or Music Performance Review or Bibliographies or Biographical-Items or Fiction, Creative Prose or Retracted Publications or TV Review, Radio Review Videos or Abstract of Published Items or Excerpts or Software Reviews or Hardware Reviews or Items About an Individual or Item Withdrawal or Music Score or Music Score Review or Retractions or Theater Reviews (Exclude – Document Types))**
#3	**#2 NOT TS=((adolescent* OR child* OR schoolchild* OR infant* OR girl* OR boy* OR teen OR teens OR teenager* OR youth* OR pediatr* OR paediatr* OR puber*) NOT (adult* OR man OR men OR woman OR women))**
#2	**#1 AND LA=(dutch OR english OR french OR german)**
#1	**TS=(vertig* OR dizz* OR vestibul*)**

covidence

Systematic Review VERDI-study

Figure 1

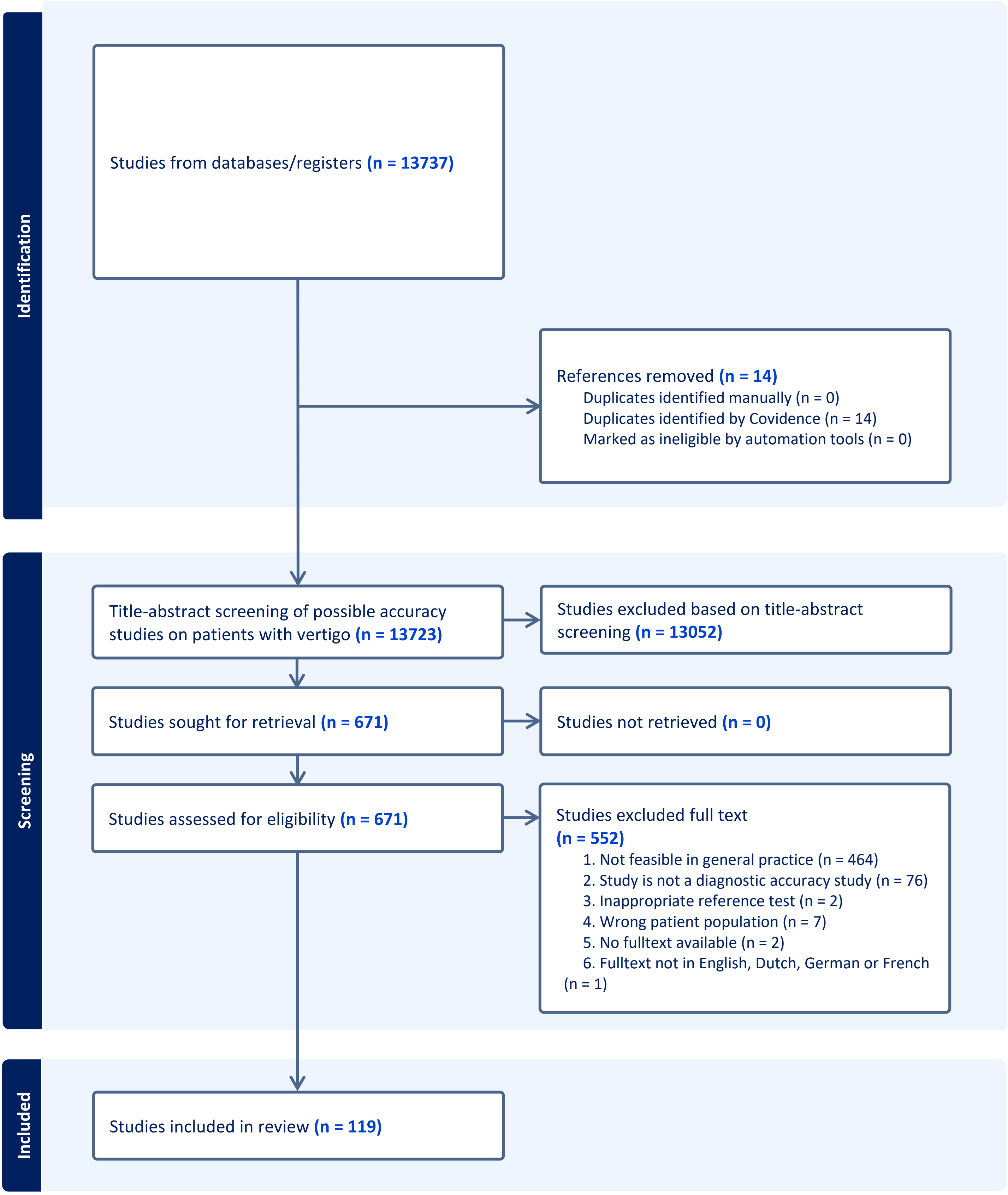

References

René Otten, Ralph de Vries, & Linda Schoonmade. (2019). Amsterdam Efficient Deduplication (AED) method (Version 1). Zenodo. https://doi.org/10.5281/zenodo.3582928

Lobbestael, G. (2023). DedupEndNote (Version 1.0.1 20240114) [Computer software]. https://github.com/globbestael/DedupEndNote

covidence
